# Root-knot nematode assessment: species identification, distribution, and new host records in Portugal

**DOI:** 10.3389/fpls.2023.1230968

**Published:** 2023-08-08

**Authors:** Leidy Rusinque, Maria João Camacho, Clara Serra, Filomena Nóbrega, Maria L. Inácio

**Affiliations:** ^1^ Instituto Nacional de Investigação Agrária e Veterinária (INIAV, I.P.), Oeiras, Portugal; ^2^ Centre for Functional Ecology (CEF), Department of Life Science, University of Coimbra, Coimbra, Portugal; ^3^ Chemical Process Engineering and Forest Products Research Centre (CIEPQPF), Department of Chemical Engineering, University of Coimbra, Coimbra, Portugal; ^4^ NemaLab, MED – Mediterranean Institute for Agriculture, Environment and Development, Institute for Advanced Studies and Research, University of Évora, Évora, Portugal; ^5^ Direção-Geral de Alimentação e Veterinária, DGAV, Lisboa, Portugal; ^6^ GREEN-IT Bioresources for Sustainability, ITQB NOVA, Oeiras, Portugal

**Keywords:** esterase, horticulture, *Meloidogyne*, management, frequency

## Abstract

Considered one of the most devastating plant parasitic nematodes worldwide, *Meloidogyne* spp. (commonly known as the root-knot nematodes (RKNs)) are obligate sedentary endoparasites that establish in the roots, causing hyperplasia and hypertrophy of surrounding cells, triggering the formation of galls. These galls will affect root development and physiology, leading to substantial yield losses. During 2017–2022, an extensive survey of *Meloidogyne* species was undertaken in Portugal (mainland and islands). A total of 1,071 samples were collected by the National Plant Protection Organization (DGAV) and private farmers from different regions of the country and were analysed at the Laboratory of Nematology (NemaINIAV). Samples in which the presence of *Meloidogyne* sp. was detected were used to perform bioassays to obtain females and juveniles for further studies. Since the accurate identification of RKNs is an important aspect of crop management, morphological and biochemical characterisation was performed. The most common morphological features were observed, showing consistency with previous descriptions of the genus. The biochemical identification using the esterase (EST) phenotype revealed the phenotypes of *Meloidogyne arenaria*, *M enterolobi*, *M. hispanica*, *M. hapla*, *M. incognita*, *M javanica*, and *M. luci. Meloidogyne incognita* and *M. javanica* were found to be the most prevalent species in the different regions followed by *M. arenaria* and *M. hapla*. This is the first distribution report performed in Portugal on RKNs, contributing to the development of management strategies and to updated information on the status of these pests in Europe.

## 1 Introduction

Agriculture is the practice of cultivating natural resources to sustain human life and generate profits. In the EU, agricultural production is a big business, contributing EUR 217 billion towards the EU’s overall gross domestic product (GDP) in 2022, and it is expected to grow due to the increase in global trade caused by the growing population. European countries contribute to the total output value of the EU’s agricultural industry, being more than half (56.9%) coming from France, Germany, Italy, and Spain. Portugal contributes less than 5% (Eurostat, 2023).

In territorial, social, and economic terms, agriculture in Portugal has great importance for the whole country but particularly to rural areas concerning sustainable development. It is also well positioned in the European and world markets due to its climate, biodiversity, innovation, and ability to present differentiated and safe products. According to data from Instituto Nacional de Estatística’s economic accounts, it appears that in 2018, vegetable and horticultural products represented 17% of the national agricultural production, of which fresh vegetables represent 50% of production (GPP, 2020; INE, 2022). According to PORDATA, agriculture and forestry have an essential role in preserving the environment and landscapes in Portugal; together, they cover 75.3% of the land. Moreover, approximately 26.2% of the agricultural area corresponds to arable land, which is divided into perennial crops (21.7%), pastures (51.7%), and family farming (0.4%).

Portugal is divided into seven regions (North, Metropolitan area–Lisbon, Centre, Alentejo, Algarve, Madeira, and Azores), which have different regional specialisations because of the considerable diversity of natural and economic–social conditions (Avillez, 2015; Freire and Lains, 2017). The regions of Alentejo and Azores have the highest significance in the national agriculture production, representing 8.6% and 6.8% of GDP, respectively (Marques, 2015; GPP, 2020).

Plant parasitic nematodes (PPNs) are regarded as one of the most important soil-borne pests, accounting for USD 175 billion per year in yield losses worldwide (Bernard et al, 2017). One of the oldest and most economically important PPNs are the root-knot nematodes (RKNs), *Meloidogyne* spp., which are considered serious pests for agricultural production, causing annual losses of USD 157 billion globally (Abad et al., 2008). This genus comprises more than 100 species (Subbotin et al, 2021); species *Meloidogyne arenaria* (Neal, 1889) Chitwood, 1949, *Meloidogyne hapla* Chitwood, 1949, *Meloidogyne incognita* (Kofoid and White, 1919) Chitwood, 1949, and *Meloidogyne javanica* (Trub, 1885) Chitwood, 1949, are known as the most important due to their widespread distribution and broad host range (Jones et al., 2013). In Portugal, so far, only 10 species have been reported (**Table 1**).

**Table 1:** Species of *Meloidogyne* sp. detected in Portugal.

Species	Author(s)
*Meloidogyne arenaria* (Neal, 1889)	Pais and Abrantes, 1989
*Meloidogyne chitwoodi* (Golden et al., 1980)	Da Conceição et al., 2009
*Meloidogyne enterolobii* (Yang and Eisenback, 1983)	Santos et al., 2019
*Meloidogyne hapla* (Chitwood, 1949)	Pais and Abrantes, 1989; Abrantes et al., 2008
*Meloidogyne hispanica* (Hirschmann, 1986)	Pais and Abrantes, 1989; Abrantes et al., 2008
*Meloidogyne incognita* (Kofoid and White, 1919) Chitwood, 1949	Pais and Abrantes, 1989; Abrantes et al., 2008
*Meloidogyne javanica* (Trub, 1885) Chitwood, 1949	Pais and Abrantes, 1989
*Meloidogyne luci* Carneiro et al, 2000	Maleita et al., 2018; Rusinque et al., 2021
*Meloidogyne lusitanica* (Abrantes and de A. Santos, 1991)	Abrantes and de A. Santos, 1991
*Meloidogyne naasi* (Franklin, 1965)	Viera dos Santos et al., 2020

Root-knot nematodes infect at the elongation zone and then move to the root tips to invade the vascular cylinder and form a feeding site, called a giant cell. At the same time, the neighbouring cells start to divide to form the typical gall or root-knot, affecting the development of the root system and causing significant yield losses (Jena and Rao, 1973; Norton and Niblack, 1991; Kyndt et al., 2014).

Most RKN species have high plasticity, enabling their establishment in different geographical areas and colonisation of different hosts. Moreover, projections by the intergovernmental panel for climate change indicate that the elevated temperature and moisture may result in an increasing rate of infection, development, and reproduction, causing shifts in their abundance and geographic distribution (Mbow et al., 2019).

Considering the impact that the RKNs have on agricultural production, species identification is essential to define sustainable management strategies. Morphological RKN identification is a valuable tool with low cost and accuracy depending on the number of characteristics and specimens evaluated. Furthermore, the biochemical electrophoretic analysis of non-specific esterase (EST), along with several molecular methods, such as internal transcribed spacer–polymerase chain reaction–restriction fragment length polymorphism (ITS-PCR-RFLP), sequence characterized amplified region (SCAR) markers, real-time PCR, and loop-mediated isothermal amplification (LAMP), have proved to be useful in the differentiation of economically important species of *Meloidogyne*.

Currently, in Portugal, there is a lack of detailed information on the root-knot nematode geographical distribution and species occurrence. Therefore, this study aimed to assess the presence and incidence of the RKNs in Portugal, thus contributing to knowledge about the wide dissemination of these nematodes and designing and implementing effective management practices.

## 2 Materials and methods

### 2.1 Sampling

**Figure 1:**
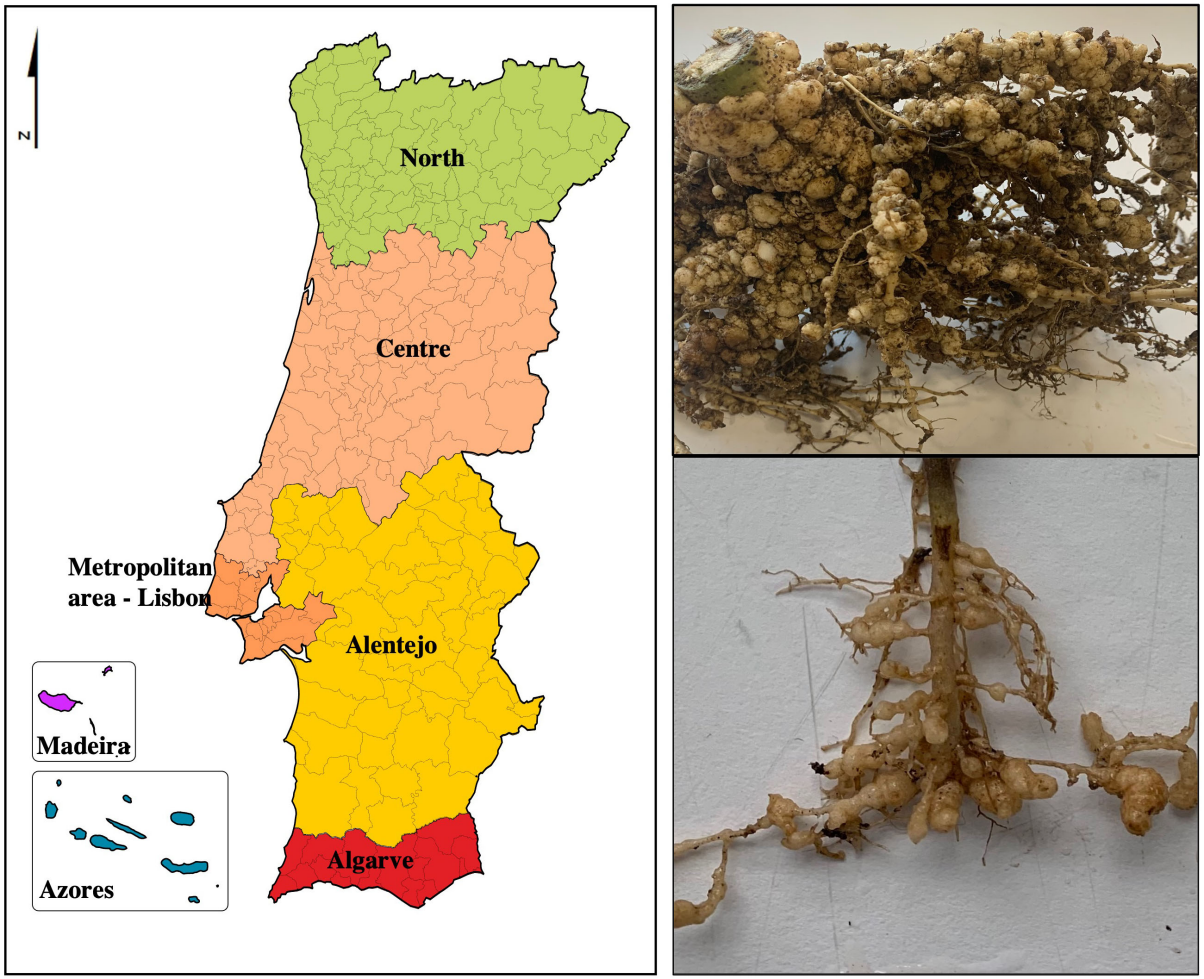
Regions of Portugal and symptoms of root-knot nematodes (*Meloidogyne* sp.) in (host) root.

From 2017 to 2022, soil and root samples were collected by inspectors of the National Plant Protection Organization (DGAV-Portugal) and by private farmers from the different regions of Portugal (**Figure 1**; **Table 2**). Samples with a volume of 1,500 mL of soil/ha were collected from the rhizosphere at approximately 15–20-cm depth for horticultural crops and 90 cm for trees. At least 100 subsamples/ha were harvested in a rectangular mesh, not less than 5 m wide and no more than 20 m long between sampling points, covering the entire field. Samples were stored in polyethylene bags and individually coded. Geographical locations at district and county levels as well as the crops installed in these fields were accessed only after the result analysis.

**Table 2:** | Number of samples collected in each Portuguese region between 2017 and 2022.

Region	2017	2018	2019	2020	2021	2022	Total/region
North	32	33	17	11	15	23	130
Centre	40	42	59	122	70	27	360
Metropolitan area–Lisbon	5	32	12	21	9	23	102
Alentejo	14	28	78	70	55	49	293
Algarve	7	10	21	14	11	12	77
Azores Island	—	3	18	15	49	20	105
Madeira Island	—	—	—	3	—	1	4
Total per year	98	148	205	256	209	155	1,071

Carrot, potato, and tomato were the main crops surveyed; however, samples from other crops such as broccoli, cabbage, chard, courgette, cucumber, orange tree, spinach, strawberry, and sweet potato were included.

### 2.2 Nematode extraction

Nematodes were extracted from a 500-mL subsample using the Oostenbrink dish technique according to protocol PM 7/119 (1) (Standard Protocol PM 7/119 (1), 2013). The suspensions were observed under a stereomicroscope (Nikon SMZ1500, Tokyo, Japan), and suspected specimens of *Meloidogyne* sp. were observed using a bright-field light microscope (Olympus BX-51, Hamburg, Germany) for confirmation. Roots were also examined for gall presence.

### 2.3 Morphological characterisation

Morphological characterisation was performed using second-stage juveniles (J2) and males individually placed in a drop of water on a glass slide and gently heat killed. Nematodes were observed using a bright-field light microscope (Olympus BX-51, Hamburg, Germany) and photographed with a digital camera (Leica MC190 HD, Wetzlar, Germany). The features observed for characterisation were stylet, excretory pore, tail, hyaline tail terminus, and spicule. Additionally, perineal patterns of mature females were cut in 45% lactic acid and permanently mounted in glycerin (Hartman and Sasser, 1985).

### 2.4 Biochemical characterisation

Bioassays were carried out by planting tomato plants cv. Oxheart in the remaining soil or inoculating them using egg masses and maintaining them in a quarantine greenhouse for 2 months to obtain material for further studies. From infected tomato roots, young egg-laying females were handpicked and transferred to micro-haematocrit capillary tubes (one female per tube) with 5 µL of extraction buffer (20% sucrose v/v and 1% Triton X-100 v/v). Maceration of the females was performed with a pestle, frozen, and stored at −20°C until use. After centrifugation, the protein extracts were separated by polyacrylamide gel electrophoresis (PAGE) on thin-slab 7% separating polyacrylamide gels in a Mini-Protean II (BioRad Laboratories, Hercules, CA, USA) according to Esbenshade (1985) and Pais et al. (1986). The gels were stained for EST activity with the substrate α-naphthyl acetate. Protein extracts of *Meloidogyne javanica* (Treub, 1885) isolate were included in the gel as a reference.

### 2.5 Statistical analysis

To evaluate the frequency and abundance of the different species of *Meloidogyne* in Portugal, multiple proportion tests were performed using the software R (https://www.r-project.org). For one of the tests, only samples identified to species level were used. The hypothesis tests were performed with a significance level α = 0.05.

*Meloidogyne* sp.-positive detections maps were made using the ArcMap 10.6 software (ESRI, USA): CAOP2017_PORTUGAL and CAOPP2017_DISTRITOS shapefiles (DGT, 2017) for continental detections, CAOP2019_Madeira shapefiles (DGT, 2019a) for Madeira Island detections, and CAOP2019_Açores (Grupo Oriental), CAOP2019_Açores (Grupo Central), and CAOP2019_Açores (Grupo Occidental) shapefiles for Azores Island detections (DGT, 2019b; DGT, 2019c; DGT, 2019d).

## 3 Results and discussion

**Figure 2:**
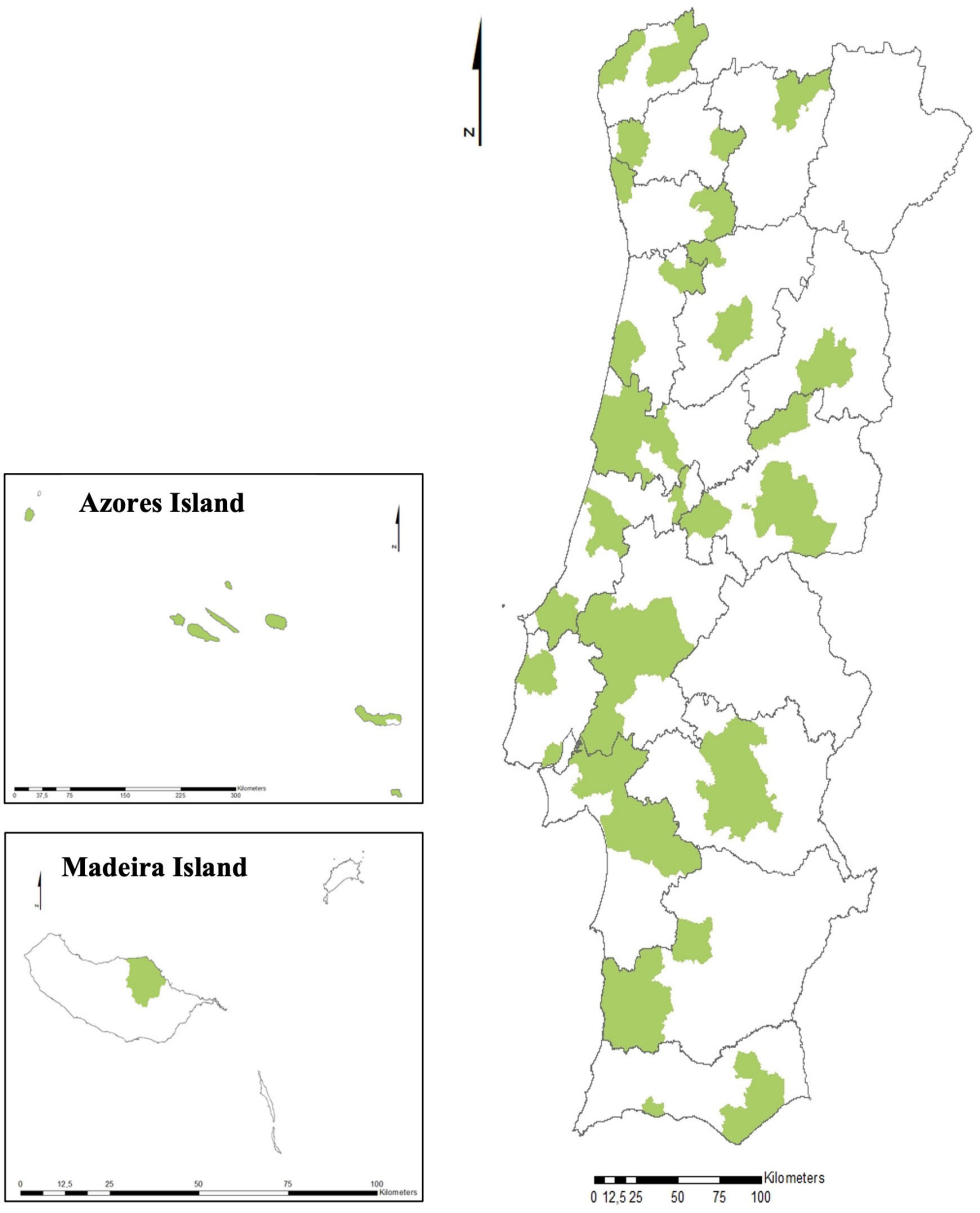
Positive detections of *Meloidogyne* sp. in Portugal (mainland and islands), 2017–2022.

During the period 2017–2022, a total of 1,071 samples were collected and analysed from the seven regions of Portugal. Root-knot nematodes were detected in 243 samples distributed along the country (mainland and islands) and corresponding to 22.7% of the total (**Figure 2**). Among the positive detections, Azores Island contributed with 37% (90 samples), followed by the region of Alentejo at 22.7% (55 samples) and the Centre at 18.5% (45 samples) (**Table 3**).

**Table 3:** Positive and negative detections of *Meloidogyne* in the seven Portuguese regions between 2017 and 2022 (absolute values and %).

Region	Positive detections		Negative detections	
	Number of samples	%*	Number of samples	%*
North	21	8.6	109	13.1
Centre	45	18.5	315	38
Metropolitan area Lisbon	13	5.4	89	10.8
Alentejo	55	22.7	238	28.8
Algarve	16	6.6	61	7.4
Azores Island	90	37	15	1.8
Madeira Island	3	1.2	1	0.1
Total	243	22.7	828	77.3

* Corresponds to the percentage out of the total of positive/negative samples.

The statistical analysis confirmed that the abundance of *Meloidogyne* is not equal in all regions, and comparison tests at a significance level of 5% showed that the region of Azores has a significantly higher abundance than the rest of the regions.

**Figure 3:**
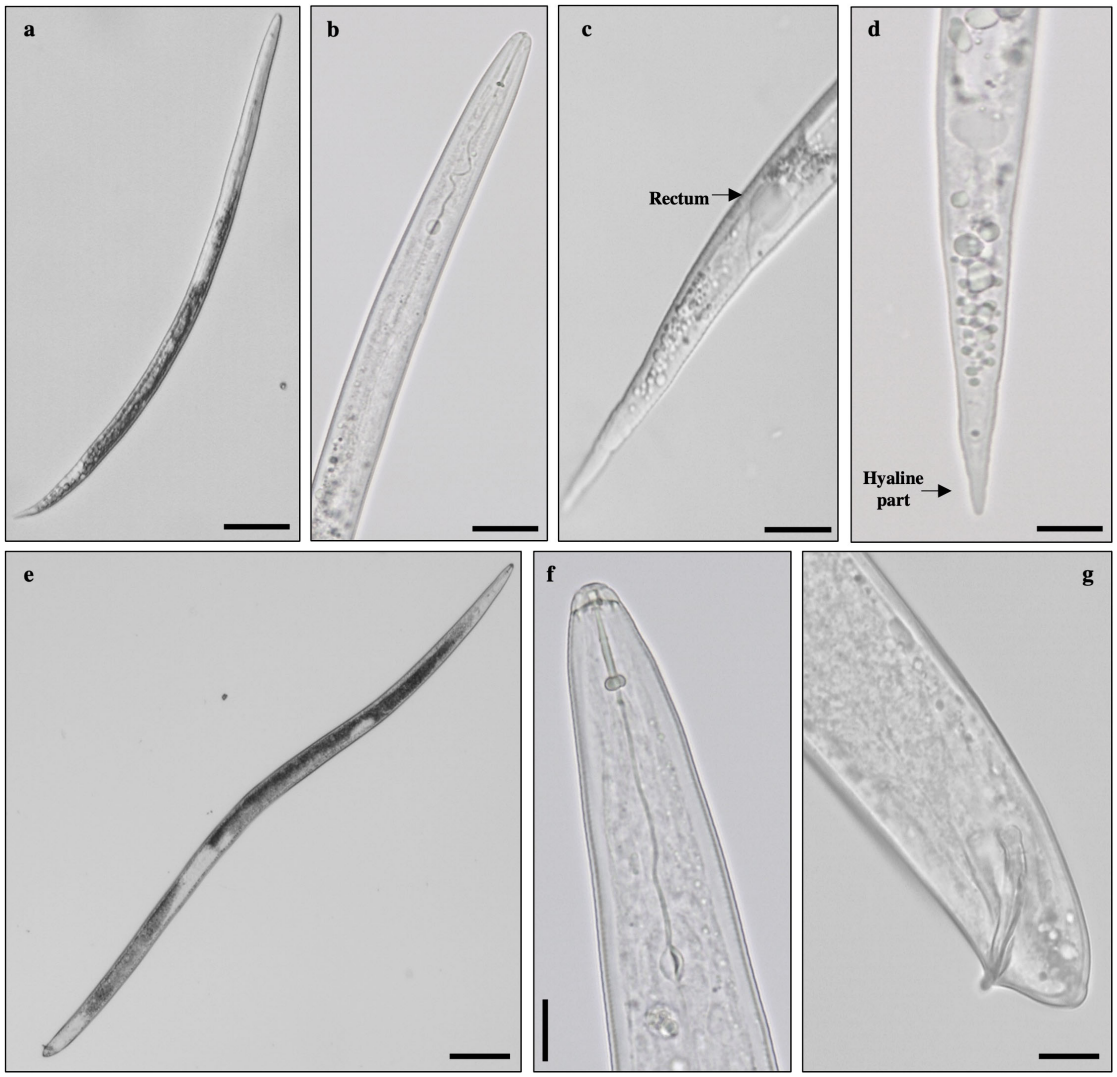
Morphological traits observed in specimens of *Meloidogyne* sp. Second-stage juvenile: **(A)** whole specimen, **(B)** anterior region, **(C)** tail region–rectum, **(D)** tail region–hyaline part. Male: **(E)** whole specimen, **(F)** head region, and **(G)** spicule. Bar = 20 µm.

Morphological characterisation of second-stage juveniles recovered from soil was performed on 10 specimens. Nematodes were vermiform, slender, and annulated. The head region was slightly set off from the body. The stylet was delicate, narrow, and sharply pointed, with small knobs. The excretory pore was distinct. The tail was conoid with a hyaline terminus distinctive in most species. Males were vermiform, bluntly rounded posteriorly and with an anterior end narrowing. The head region was smooth, not set off from the body. The stylet was robust, with a straight cone, pointed and widen gradually to the posterior end. Knobs were rounded merging gradually into the shaft. The tail was short and round. Spicules were long and curved (**Figure 3**), agreeing with previous descriptions from Eisenback (1985) and Jepson (1987). Some specimens presented vesicle-like structures around the lumen of the juvenile metacorpus characteristic of *Meloidogyne naasi* and *Meloidogyne sasseri*. Therefore, to determine the identity of these specimens, as stated by Karssen (1996), morphometrics of second-stage juveniles (body, tail, and hyaline tail terminus length) were carried out, confirming the presence of *M. naasi*.

**Figure 4:**
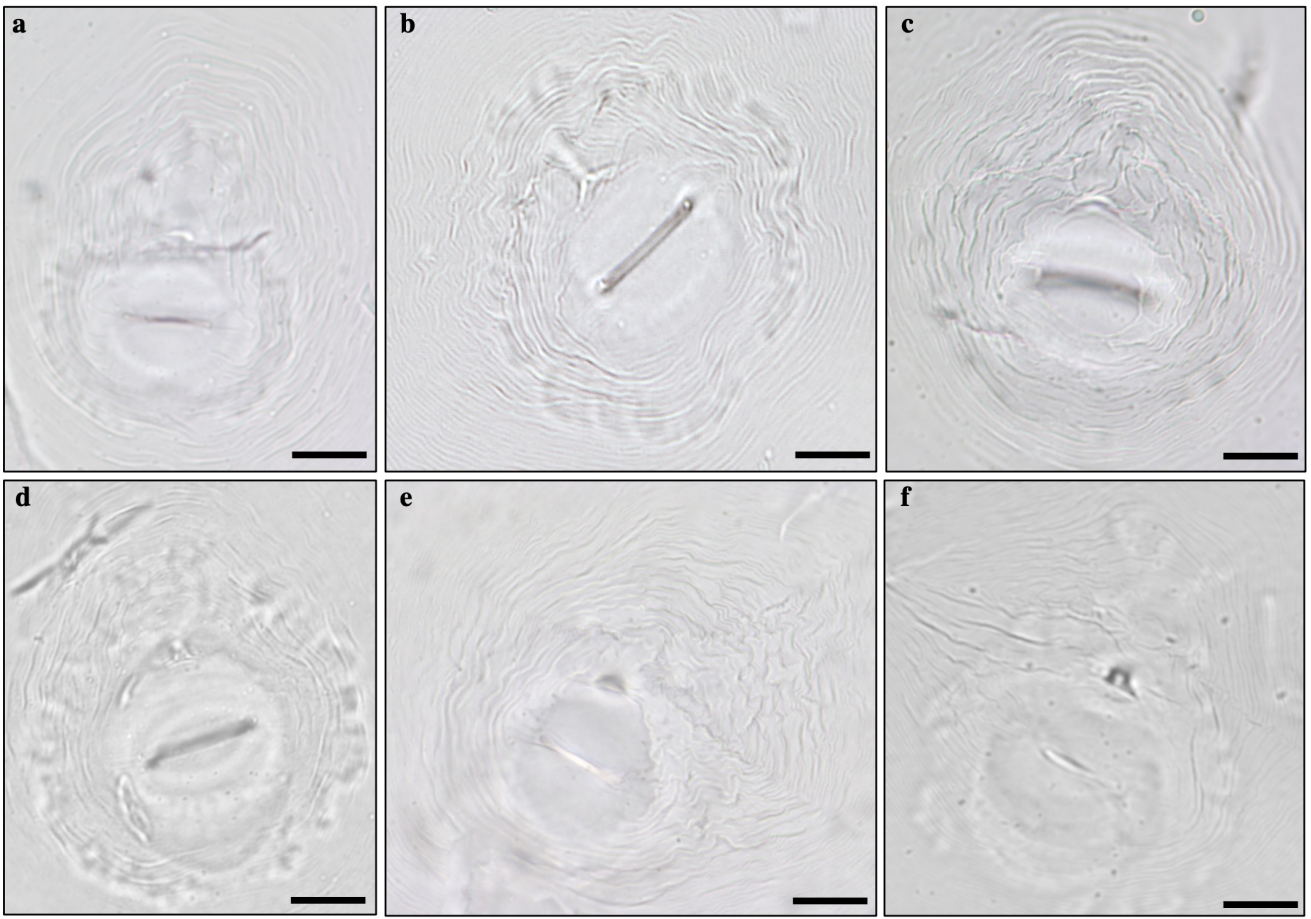
Perineal patterns observed in specimens of *Meloidogyne* sp.: **(A)** *Meloidogyne arenaria*, **(B)** *Meloidogyne incognita*, **(C)** *Meloidogyne javanica*, **(D)** *Meloidogyne enterolobii*, **(E)** *Meloidogyne luci*, and **(F)** *Meloidogyne hispanica*. Bar = 20 µm.

Females were elongated, ovoid, or pear-shaped. The perineal pattern comprised the vulva-anus area, tail terminus, phasmids, lateral lines, and surronding cuticule striae (Karssen et al., 2013). Some variability was observed among the perineal patterns. The shape was ovoid to rounded in the species *Meloidogyne arenaria*, *M. incognita*, *M. javanica*, and *M. enterolobii* while oval to squarish in species *M. luci* and *M. hispanica*. Distinctive lateral lines were present in *M. javanica* and *M. arenaria* whereas absent or weakly demarcated in *M. incognita*, *M. luci*, and *M. enterolobii* (**Figure 4**).

**Figure 5:**
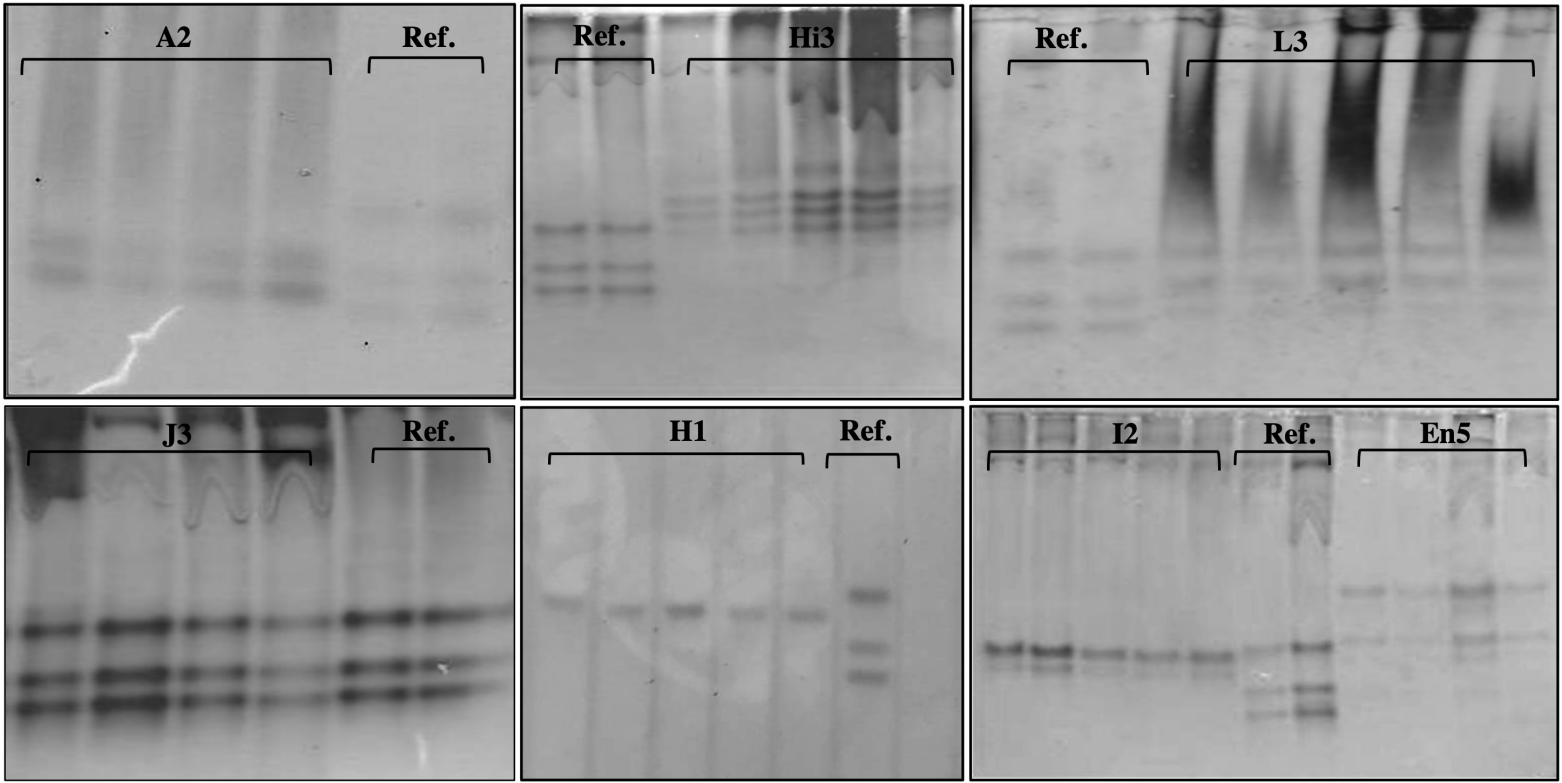
Esterase phenotypes of protein homogenates from one egg-laying female of Meloidogyne species: *Meloidogyne arenaria* (A2), *Meloidogyne hispanica* (Hi3), *Meloidogyne luci* (L3), *Meloidogyne javanica* (J3), *Meloidogyne hapla* (H1), *Meloidogyne incognita* (I2), *Meloidogyne enterolobii* (En5), and reference (J3).

Enzyme phenotype analyses allowed us to identify some of the species present within the positive samples. Despite many attempts, it was only possible to reach the species identification of 51% of the positive samples (123). For the remaining 49%, we can only confirm the presence of individuals of the genus *Meloidogyne*. Seven different phenotypes were identified corresponding to the following species of root-knot nematodes: *Meloidogyne arenaria*–A2 phenotype, *M. enterolobii*–En5 phenotype, *M. hapla*–H1 phenotype, *M. hispanica*–Hi3 phenotype, *M. incognita*–I2 phenotype, *M. javanica*–J3 phenotype, and *M. luci*–L3 phenotype (**Figure 5**).

The host status of crops of economic importance and the host range of *Meloidogyne* spp. are issues of major concern in integrated nematode management recommendations. Currently, in Portugal, this information is very incomplete. In this survey, RKN species were found parasitising 22 different plant hosts, mostly horticultural crops in open fields and also grasses, ornamentals, and fruit trees (**Table 4**). So far, many of these crops have not been reported in Portugal as hosts of *Meloidogyne*. Therefore, to our knowledge, this is the first report of species of *Meloidogyne* parasitising aubergine, broccoli, carrot, chard, courgette, orange tree, okra, pepper, and strawberry in Portugal’s mainland.

**Table 4:** Crops and regions associated with each species of *Meloidogyne* sp. identified in Portugal.

Species identified	Region	Host plant	Cultivation system
*Meloidogyne arenaria* (10 samples = 8.1%)	Azores Island	Green beans (*Phaseolus vulgaris*)	Greenhouse
	Alentejo	Aubergine (*Solanum melongena*)	Open field
	Azores Island	Cabbage (*Brassica oleracea*)	
	Centre	Cabbage tree (*Cordyline australis*)	
	Algarve	Orange tree (*Citrus sinensis*)	
	North, Algarve, and Azores Island	Potato (*Solanum tuberosum*)	
*Meloidogyne incognita* (71 samples = 57.8%)	Centre and Azores Island	Broccoli (*Brassica oleracea* cv. *italica*)	Greenhouse
	Alentejo and Azores Island	Courgette (*Cucurbita pepo*)	
	Metropolitan area–Lisbon and Azores Island	Cucumber (*Cucumis sativus*)	
	Azores Island	Pea (*Pisum sativum*)	
	Alentejo and Azores Island	Pepper (*Capsicum annuum*)	
	Metropolitan area–Lisbon	Carrot (*Daucus carota* subsp. *sativus*)	Open field
	Metropolitan area–Lisbon and Azores Island	Chard (*Beta vulgaris* subsp. *vulgaris*)	
	Azores Island	Leek (*Allium porrum*)	
	Azores Island	Onion (*Allium cepa*)	
	Alentejo	Okra (*Abelmoschus esculentus*)	
	North, Centre, and Azores Island	Potato (*S. tuberosum*)	
	North, Alentejo, Algarve, and Azores Island	Tomato (*Solanum lycopersicum*)	Greenhouse and open field
*Meloidogyne javanica* (20 samples = 16.2%)	Azores Island	Spinach	Greenhouse
	Centre Centre Centre	Carrot (*D. carota* subsp. *sativus*)	Open field
		Cabbage tree (*C. australis*)	
		Grapevine (*Vitis vinifera*)	
	Alentejo	Okra (*A. esculentus*)	
	Alentejo and Azores Island	Potato (*S. tuberosum*)	
	Alentejo and Algarve	Tomato (*S. lycopersicum*)	
*Meloidogyne hapla* (12 samples = 9.7%)	Centre	Cabbage tree (*C. australis*)	Open field
	Alentejo	Eucalyptus (*Eucalyptus globulus*)	
	Metropolitan area–Lisbon and Madeira Island	Grapevine (*V. vinifera*)	
	Alentejo	Pepper (*C. annuum*)	
	North	Potato (*S. tuberosum*)	
	Centre	Strawberry (*Fragaria* × *ananassa*)	
	North	Tomato (*S. lycopersicum*)	
*Meloidogyne enterolobii* (1 sample = 0.8%)	Centre	Cabbage tree (*C. australis*)	Open field
*Meloidogyne luci* (4 samples = 3.3%)	Centre and Azores Island	Potato (*S. tuberosum*)	Open field
*Meloidogyne hispanica* (4 samples = 3.3%)	Centre, Algarve and Azores Island	Potato (*S. tuberosum*)	Open field
*Meloidogyne naasi* (1 sample = 0.8%)	Metropolitan area–Lisbon	Turfgrass	Open field

Total of samples identified to species level (123 = 51%).

The statistical analysis regarding the different species of RKNs showed that *M. incognita* and *M. javanica* presence was significantly different (p-value ≤2e−16) from the rest of the species, indicating that the frequency of occurrence in fields across the country is high. The less frequent species found in the fields were *M. enterolobii* and *M. naasi* with a p-value ≤0.77.

**Figure 6:**
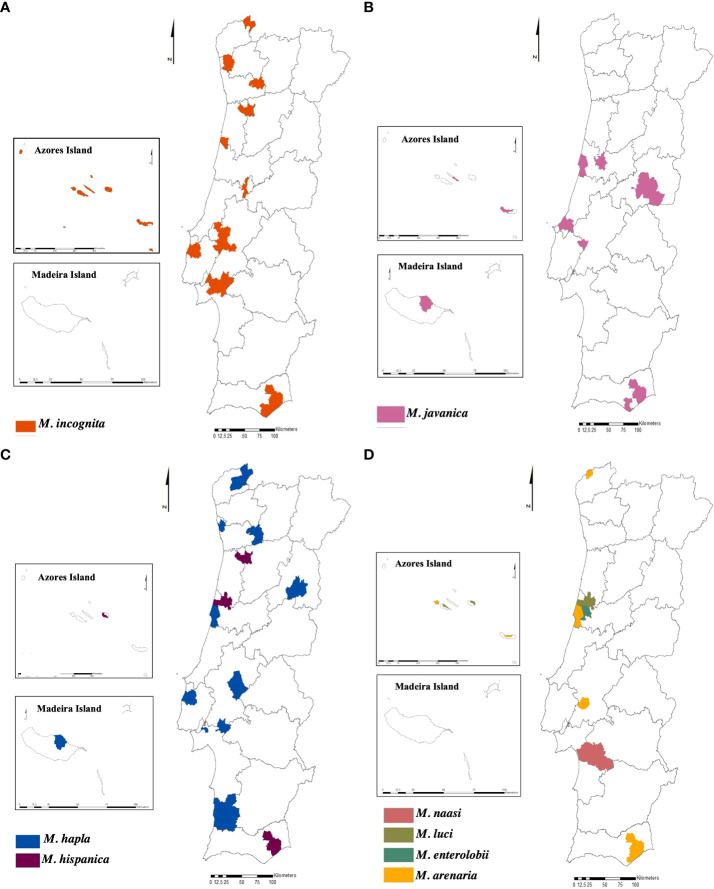
Distribution of the species of *Meloidogyne* sp. in Portugal (mainland and islands). **(A)** *Meloidogyne incognita*. **(B)** *Meloidogyne javanica*. **(C)** *Meloidogyne hapla* and *Meloidogyne hispanica*. **(D)** *Meloidogyne naasi, Meloidogyne luci, Meloidogyne enterolobii*, and *Meloidogyne arenaria*.

From the total samples identified to species level, *M. incognita* is the predominant species in the country (mainland and islands), as it was found in 71 samples (57.8%) and six of the seven regions surveyed. Following in prevalence, *M. javanica* was identified in 20 samples (16.2%) and present in five regions. *Meloidogyne arenaria* and *M. hapla* were detected in 10 samples (8.1%) and 12 samples (9.7%), respectively, and were present in four regions. Species of the least frequent occurrence “minor species” such as *M. enterolobii*, *M. hispanica*, *M. luci*, and *M. naasi*, were also detected, corresponding to 8.2% of the total. The region with the highest diversity of species is the centre region (seven species) followed by the Azores Island (five species), Alentejo, Algarve (four species), and North (three species) (**Figures 6A–D**).

Plant parasitic nematodes represent a risk to agricultural production worldwide. Once a field is infested, it is difficult to eradicate them. Instead, the goal is to keep nematode densities low and reduce crop damage. RKNs are among the most widely distributed pests causing economically important damage to a great range of crops. Due to their importance and given the current concerns regarding climate change and food security, the main challenge is to find management strategies that can be efficient and sustainable to control them since the current practices are not enough; therefore, species identification is essential.

Although traditionally morphology is used for RKN identification, currently, it represents a challenge due to the variability between individuals, the indistinctive differences among them, and the increase in the number of species (Eisenback, 1985; Hirschmann and Volume, 1985; KarssenVan Aelst, 2001). Hence, it is of primary importance to have specialised and well-trained researchers to minimise the level of inaccuracy.

Furthermore, the effectiveness of the non-specific EST phenotype as the more stable and quicker method to identify *Meloidogyne* spp. has been demonstrated in many studies, showing to be highly polymorphic and able to detect different EST phenotypes of a single female (Esbenshade and Triantaphyllou, 1985; Carneiro et al., 2000). Nonetheless, its main disadvantage is that requires females in a specific developmental stage (Hunt and Handoo, 2009).

Among the tropical species found in this study, *M. incognita*, *M. arenaria*, and *M. javanica* are probably the most widely distributed and economically important species of plant parasitic nematodes, so much that in some areas of the world, galls on roots are considered normal. The species *M. luci* is included in the European and Mediterranean Plant Protection (EPPO) alert list and *M. enterolobii* in the A2 List of pests recommended for regulation as quarantine pests (EPPO, 2017a). Their habitats in general terms are the tropical and subtropical regions; however, they have also been found in temperate zones overwintering in mild winters. Meanwhile, *M. hapla* occurred mainly in temperate regions, being able to survive in temperatures below 0°C, though there is no evidence of its inability to survive in hot temperatures. Based on the above, the presence of these species in Portugal in a wide variety of hosts and climates is not an unusual event; on the contrary, it is an expected fact since the temperature increase is contributing to the geographical expansion not only of these major species but also of species of minor or restricted occurrence.

Management of RKNs is difficult due to the complexity of the soil environment (Norton and Schmitt, 1978). Biological, cultural, and chemical methods are some of the strategies that have reduced the risk of damage by many nematode species (Hague and Gowen, 1987; Heald, 1987; Kerry, 1987; Halbrendt and La Mondia, 2004; Starr and Roberts, 2004). However, all these techniques have associated challenges (Abd-Elgawad, 2022a). Synthetic nematicides were a commonly used strategy; nevertheless, some active substances have been strictly regulated or banned from the market owing to adverse environmental and health impacts, reducing the number of alternatives for control.

Cultural methods also appear to control to some degree RKNs; however, the extensive host range that includes nearly every horticultural, fruit, and ornamental crop poses severe constraints. Similarly, many bacterial and fungal agents as well as chemical compounds have been described for *Meloidogyne* spp. as a potential strategy to be included in integrated pest management programs, among which some have not yet been tested in the field and others have not provided consistent results (Faria et al., 2022; Pires et al., 2022). Resistant cultivars have also shown some efficacy on RKN control, but some species are able to overcome that resistance and the cultivars are not always commercially available (Abd-Elgawad, 2022b). A combination of microbial strategies using both bacterial and fungal agents with other cultural control practices or host resistance poses an alternative that can be used as a multidisciplinary approach to improve the management strategies for RKNs.

Finally, extensive surveys had not been performed in Portugal, and so, the results presented here confirm other reports on the widespread distribution of *Meloidogyne*, its high frequency of occurrence, and its potential as a problem for agricultural production. This assessment included crops of economic importance that are grown, intensively favouring the survival and rapid build-up of nematode populations in the soil. This fact and the ability of RKNs to be transmitted by soil, agricultural machinery, infected plants, and running water explain the presence of a high number of species in a wide diversity of hosts. The information here presented regarding the species of *Meloidogyne* found in the country will help farmers and technicians in the development and establishment of efficient and sustainable practices and policymakers in the provision of phytosanitary measures and monitoring programmes to prevent the introduction and spread of these pests of concern in Europe.

## 4 Conclusion

This study shows the high occurrence and frequency of RKNs in Portugal, confirming the widespread distribution of these nematodes. Moreover, the detection of a great variety of species of *Meloidogyne* in different regions around the country evidenced that there is a northward movement of pests caused by trade activity and climate changes. Due to this fact, the identification of *Meloidogyne* species is of great importance for the development of appropriate management practices for its control.

## Data availability statement

The original contributions presented in the study are included in the article/supplementary material, further inquiries can be directed to the corresponding author.

## Author contributions

Conceptualisation: LR. Research and data analysis: LR, MC, MI, and FN. Writing—original draft preparation: LR. Writing—review and editing: LR, MC, CS, FN, and MI. Resources: MI. All authors contributed to the article and approved the submitted version.
